# Ethnic and racial differences in Asian populations with ion channelopathies associated with sudden cardiac death

**DOI:** 10.3389/fcvm.2023.1253479

**Published:** 2023-08-04

**Authors:** Sahil Zaveri, Yongxia Sarah Qu, Mohamed Chahine, Mohamed Boutjdir

**Affiliations:** ^1^ Department of Medicine, Cell Biology, and Pharmacology, State University of New York Downstate Health Sciences University, Brooklyn, NY, United States; ^2^ Cardiovascular Research Program, VA New York Harbor Healthcare System, New York, NY, United States; ^3^ Department of Cardiology, New York Presbyterian Brooklyn Methodist Hospital, New York, NY, United States; ^4^ CERVO Brain Research Center, Institut Universitaire en Santé Mentale de Québec, Québec, QC, Canada; ^5^ Department of Medicine, Faculté de Médecine, Université Laval, Quebec, QC, Canada; ^6^ Division of Cardiology, Department of Medicine, NYU Grossman School of Medicine, New York, NY, United States

**Keywords:** ethnicity, race, brugada syndrome (BrS), long QT syndrome (LQTS), cardiac arrhythmia, sudden cardiac death, ion channels

## Abstract

Cardiovascular diseases are associated with several morbidities and are the most common cause of worldwide disease-related fatalities. Studies show that treatment and outcome-related differences for cardiovascular diseases disproportionately affect minorities in the United States. The emergence of ethnic and racial differences in sudden cardiac death (SCD) and related ion channelopathies complicates cardiovascular disease prevention, diagnosis, management, prognosis, and treatment objectives for patients and physicians alike. This review compiles and synthesizes current research in cardiac ion channelopathies and genetic disorders in Asian populations, an underrepresented population in cardiovascular literature. We first present a brief introduction to SCD, noting relevant observations and statistics from around the world, including Asian populations. We then examined existing differences between Asian and White populations in research, treatment, and outcomes related to cardiac ion channelopathies and SCD, showing progression in thought and research over time for each ion channelopathy. The review also identifies research that explored phenotypic abnormalities, device usage, and risk of death in Asian patients. We touch upon the unique genetic risk factors in Asian populations that lead to cardiac ion channelopathies and SCD while comparing them to White and Western populations, particularly in the United States, where Asians comprise approximately 7% of the total population. We also propose potential solutions such as improving early genetic screening, addressing barriers affecting access to medical care and device utilization, physician training, and patient education on risks.

## Introduction

When evaluating the effectiveness of healthcare policies and services, it is crucial to give context to race and ethnicity. The health service treats individuals with unique social and genetic backgrounds. Among them, race and ethnicity have traditionally not been given priority when providing medical treatment, as they appear to be factors that should not influence the treatment an individual receives. However, although not readily apparent, these factors may affect the level of care an individual patient may expect or receive, for which a lack of awareness, competency, and tact compromises the ability of the health service to make accurate diagnoses and provide patient-centric treatment (1).

By 2030, racial and ethnic minorities are expected to make up 40% of the population in the United States (2). In that light, it is essential that we more accurately and clearly define the concepts of race and ethnicity. The concept of race is a social construct, and it involves an individual's appearance, genetics, geographical origin, and economic and political views (3). On the other hand, ethnicity highlights an individual's ancestry, history, culture, language, and religion while lacking biological aspects (3).

Within the context of healthcare, there may be race- or ethnic-specific predispositions to disease or genetic markers conveying risk. There is a need for research to account for the differences in risk assessment between racial and ethnic groups. Such a discussion is required to provide meaning and value to healthcare professionals so that they may ascertain the facts, draw conclusions, and make policy and procedural changes to improve awareness, understanding, and treatment. Therefore, it is imperative that we follow established conventions regarding terminology in the scientific literature regarding race and ethnicity since these are sensitive matters that may have broader implications beyond the medical perspective. Given the scarcity and fragmentation of information about channelopathies and related SCD in Asian populations, a thorough examination of the existing literature is timely and valuable in identifying potential gaps in the health service, designing clinical trials, and achieving treatment objectives for Asian populations (1). In the United States, Asians are one of the fastest-growing minority populations, mainly through immigration, and have grown to become a significant diaspora, at about 7% of the national population (4).

This review will strictly refer to “Asian populations” in a racial and ethnic context, applying to any individual from or with origins in the Asian continent. This includes the Far East, Southeast Asia, or the Indian subcontinent, including Cambodia, China, India, Iran, Japan, Korea, Malaysia, Pakistan, Philippine Islands, Taiwan, Thailand, and Vietnam, with the caveat that certain countries may be highlighted more often than others based on the availability of existing research (Table 1) (11). The review, while not being of a systematic nature, is a comprehensive assessment of literature from databases such as Google Scholar and PubMed to date. The keywords used for the literature search were: ethnicity, race, ion channelopathies, sudden cardiac death (SCD), Brugada syndrome (BrS), long QT syndrome (LQTS), short QT syndrome (SQTS), catecholaminergic polymorphic ventricular tachycardia (CPVT), and Asia. It will cross-examine inherited cardiovascular diseases focusing on ion channelopathies related to sudden cardiac arrest and SCD by referring to risk factors and susceptibility in the Asian population. Access to and use of appropriate treatment methods and devices, such as cardiac implants, in Asian populations will be briefly examined. Lastly, we also attempt to propose some solutions to reduce and prevent health differences in Asians. While we primarily attempt to address racial/ethnic differences in cardiovascular diseases and outcomes experienced by Asians in the United States, we also refer to literature from Asian countries to highlight research that provides genetic and disease susceptibility context for Asian populations.

**Table 1:** Sudden cardiac death in Asian subpopulations.

Study name	Study design	Study outcome
Epidemiology of Arrhythmias and Sudden Cardiac Death in Asia (5).	Literature review and meta-analysis	Asian subjects (Japan India China, Taiwan, South Korea, Thailand) receive much fewer pacemaker implantations and ICD usage compared to Western countries; Higher prevalence of BrS in Asian countries (Laos, Vietnam, Cambodia) compared to Western countries.
Sudden cardiac death in China: current status and future perspectives (6).	Meta-analysis	Significantly higher VF/VT incidence in China compared to USA; much lower ICD implantation rate in China compared to US despite similar SCD prevalence.
Sudden Cardiac Death in Mainland China: A Systematic Analysis (7).	Systematic literature review	Notable SCD incidence in China, comparable or greater than Japan or Korea. Rise in ion channelopathies reported as one of the main causes.
N-terminal pro-brain natriuretic peptide and sudden cardiac death in hypertrophic cardiomyopathy (8).	Clinical investigative analysis	N-terminal pro-brain natriuretic peptide is notable common factor in SCD diagnosis among Chinese HCM patients.
Unique clinical features and long term follow up of survivors of sudden cardiac death in an Asian multicenter study (9).	Clinical investigative analysis	Younger Taiwanese SADS patients are predisposed to SCD. Taiwanese SCD patients also had ischemic heart disease, albeit lower than in Caucasian SCD patients. LQTS represents a larger risk in Taiwanese SCD patients.
Sudden cardiac death among Iranian population: a two decades follow-up of Tehran lipid and glucose study (10).	Investigative analysis	Smoking, waist circumference, hypertension, diabetes mellitus, pulse rate and pre-existing cardiovascular disease are significantly related to SCD in Iranian patients.

BrS, brugada syndrome; HCM, hypertrophic cardiomyopathy; ICD, implantable cardioverter-defibrillator; LQTS, long QT syndrome; SADS, sudden arrhythmia death syndrome; SCD, sudden cardiac death; VF, ventricular fibrillation; VT, ventricular tachycardia.

## Health differences in race and ethnicity

**Figure 1:**
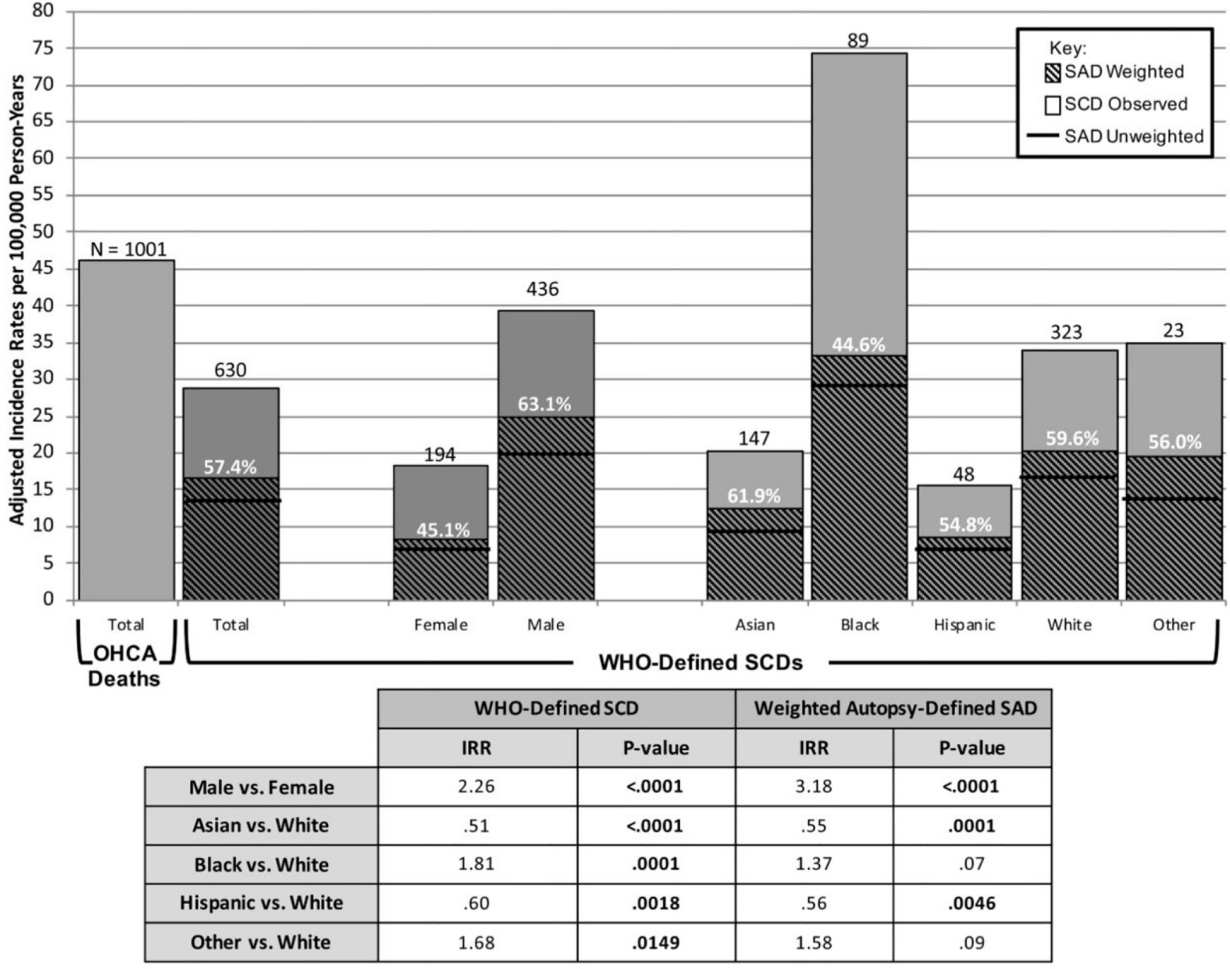
Adjusted incidence rates per 100,000 person-years for observed death groups. Adult incidence rates of out-of-hospital cardiac arrest (OHCA) death and WHO-defined sudden cardiac death (SCD) over 37 months were 46/100 000 and 29.6/100 000 person-years, respectively. OHCA death and WHO-defined SCD incidence rates include 89 identified WHO-defined SCDs and 16 OHCA deaths that did not undergo an autopsy. Incidence-rate ratios for the SCD and autopsy-defined sudden arrhythmic death (SAD) was over 2- and 3-fold higher in men vs. women, respectively (*P *< 0.0001), and lowest in Asian subjects (*P *< 0.0001). Asian (62%) had a lower proportion of SCDs that were autopsy-defined SADs than White (60%). Modified from Tseng et al. (14) with permission.

**Figure 2:**
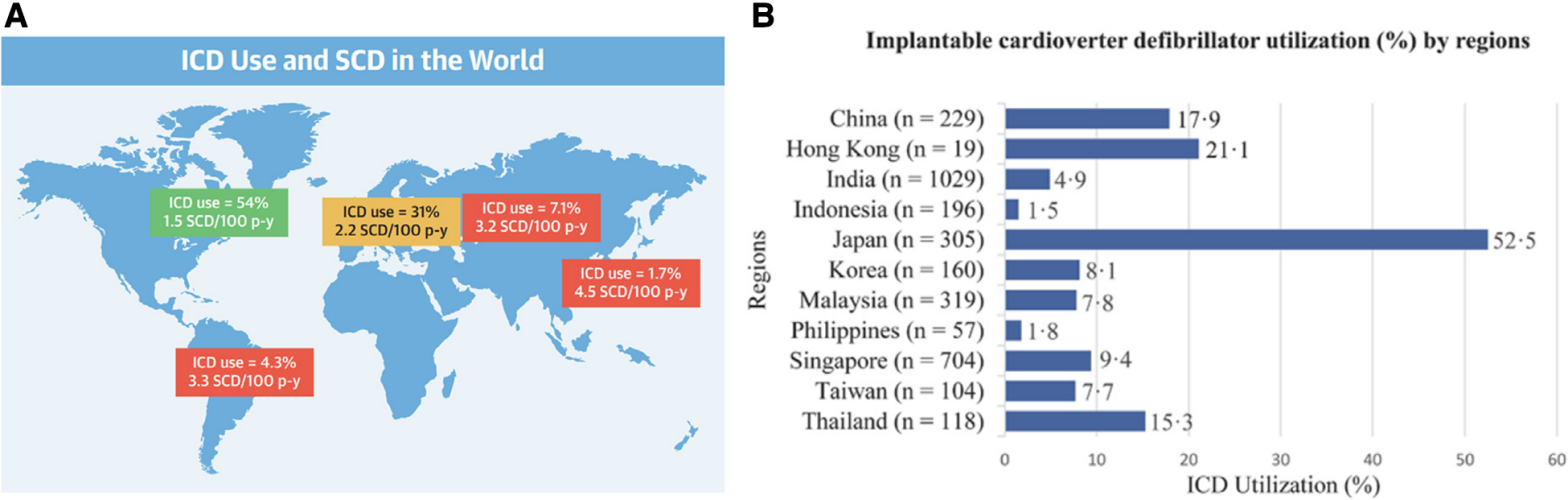
Highlighting differences in implantable cardioverter-defibrillator usage between Asia and western nations. (Panel **A**) The global landscape of implantable cardioverter-defibrillator (ICD) utilization and sudden cardiac death (SCD) rates portrays significant regional variations. Notably, North America exhibited the highest ICD usage rate (54%), while Asia-Pacific had the lowest rate (1.7%). In terms of pacemaker implantations, there were differences between Asian and White populations in various countries. (Panel **B**) ICD prevalence varied significantly across Asian countries, with Japan reporting the highest rate (52.5%), followed by Hong Kong (21.1%) and China (17.9%). A large discrepancy was observed as Indonesia reported the lowest rate (1.5%). Reprinted from Ueda et al. (15) with permission.

SCD is defined as unexpected sudden death from cardiac causes that occurs after 1 year of age and generally within 1 h from the onset of symptoms (if witnessed) or within 24 h (if the patient is alive or the event is witnessed) (5, 12). Current statistics show that 3.7 million lives are lost to SCD yearly, with over 300,000 in the US, 350,000–700,000 in Europe, and 544,000 in China (5, 6). Data show a higher SCD prevalence in men than women, owing to higher coronary artery disease prevalence in men (5). In Asia, the frequency of SCD is 37 per 100,000 people in Japan, 41 per 100,000 people in China, 38 per 100,000 people in Thailand, and 43 per 100,000 people in the Philippines (5). These statistics are lower than the 50–100 per 100,000 people incidence rates reported in Europe and the US (13). SCD incidence in Asia is 40 cases per 100,000 people annually, a significant public health concern despite being lower than in Western countries. Interestingly, Asian populations present with the highest proportion of sudden arrhythmic death to SCD when compared to Black, Hispanic, and White populations (Figure 1) (5, 14). Asia shows an increasing incidence of myocardial infarction and atrial fibrillation with age and a higher prevalence of BrS compared to Western countries, which might lead to increased mortality in young men (5). Despite implantable cardioverter-defibrillators (ICDs) being available as the primary prevention therapy, the rising cost of these devices in Asia limits access and thereby contributes to increased SCD-related mortality and morbidity rates. Recently, Ueda et al. reported that the Asia-Pacific region had the lowest ICD insertion rate of 1.7% compared to North America which had the highest ICD insertion rate of 54% (Figure 2) (15). Thus, it is imperative to prevent and reduce SCD by educating the public about its causes and risk factors (5, 15).

Since SCD is a major cause of death globally, it is important to explore different patient demographics and primary prevention therapies available for an in-depth analysis (6). Similar to Western countries and as pointed out above, SCD accounts for over 544,000 deaths per 1.3 billion individuals in China each year (6). An analysis of the ICD registry and the China Society of Pacing and Electrophysiology found a higher incidence of ventricular tachycardia and ventricular fibrillation in the Chinese population when compared to the US population; however, differing patient characteristics between the two groups raised concern about the identification of at-risk patients and SCD prevention methods, which requires further research (6). Currently, while the US has a high ICD utilization rate as the primary prevention mechanism, ICDs are underutilized in China (6). Instead, China makes greater use of arrhythmia ablation and non-ICD medical therapies, possibly due to the cost of healthcare provision (6). It is essential to recognize that optimal utilization of ICDs, in alignment with prevailing guidelines, is influenced by the economic conditions of each country as well as the characteristics of their public and/or private healthcare systems.

Exploring risk factors within affected demographics is critical to successful SCD management and prevention. A systematic review examined 41 studies conducted between 1984 and 2016 that documented a total of 6,876 cases of sudden cardiac arrest and SCD (7). The incidence rate was 40.7 per 100,000 person-years (95% CI 38.1–43.3) with a mean age of 69.5 years old, of which 55.5% of affected individuals were male (7). In the years following 2003, many causes of SCD were identified, with the most notable being the rise in cardiac ion channelopathies (0.4%) (7). The reported incidence of SCD in China, while lower than rates in the US and Europe (50–100 per 100,000 person-years), is nonetheless higher than in Japan (37 per 100,000 person-year) and South Korea (39.3 per 100,000 person-year), partly explained by differences in coronary artery disease and other risk factors in these populations (7). Cardiomyopathies were a less common cause of SCD in China compared to Western countries (10%–15%) and Japan (30%–35%), with congenital and hypertensive heart disease accounting for a higher proportion of sudden cardiac arrest and SCD (6, 7). Aging population and increased frequency of chronic conditions are noted to be common causes of SCD in the Chinese population (7, 16). Yet another study performed in 2021 on a sample of 977 Chinese patients with hypertrophic cardiomyopathy investigated the role of NT-proBNP in SCD (8). During the 3-year follow-up period, out of the original 977 patients, 29 displayed SCD, and results showed that NT-proBNP was a significant common predictor of SCD (8).

Risk factors that predispose individuals to SCD may also differ with age. Ischemic heart disease, which includes coronary artery disease and myocardial infarction, accounts for the majority of SCD incidents in older individuals (11, 17). On the other hand, inherited cardiac conditions known as sudden arrhythmic death syndrome usually predispose younger individuals to SCD. A Taiwan clinical study and follow-up (2005–2016) looked at 730 patients after a median time period of 43 months (9). The main results showed that 42% of patients in the Taiwanese SCD cohort also had ischemic heart disease, which was lower than Caucasian SCD patients. Survival studies demonstrated that sudden arrhythmic death syndrome patients had higher survival rates than those with ischemic heart disease (*P* < 0.001) (9). SCD in this particular ethnic population included a higher proportion of sudden arrhythmic death syndrome patients, a better prognosis for those with idiopathic ventricular fibrillation or idiopathic ventricular tachycardia, and a poor prognosis for individuals with LQTS (9). Individuals diagnosed with this condition that also presented channelopathies had better survival rates than those with cardiomyopathies (9). Another prospective cohort study examined 244 occurrences of SCD in a sample of Iranian men (*N* = 3,705) and women (*N* = 4,446) that were at least 30 years old after a median follow-up time of 17.9 years (10). The age-adjusted SCD incidence for men (*N* = 165) was 2.3 (95% CI 2.1–2.7) per 1,000 person-years. More than 0.2% of the Tehranian participants in this study had SCD (10). The most significant factors included ongoing smoking habits (HR 2.43, 95% CI 1.73–3.24), high waist circumference (HR 1.49, 95% CI 1.04–2.12), hypertension (HR 1.39, 95% CI 1.05–1.84), type 2 diabetes mellitus (HR 2.78, 95% CI 2.09–3.69), a pulse rate of at least 90 bpm (HR 1.72, 95% CI 1.22–2.42), and extant cardiovascular disease diagnosis (HR 1.75, 95% CI 1.26–2.45). Obesity and overweight were associated with a lower risk of SCD, showing an HR of 0.61, 95% CI 0.38–0.98, and HR of 0.58, 95% CI 0.40–0.83, respectively (10). Hypertension, smoking, diabetes mellitus, and central obesity accounted for about 65% of the Iranian SCD burden. Surprisingly, obesity was associated with a 40% lower SCD risk (10). While ICD has a proven role in preventing SCD, traditional risk factors can be targeted to address its rise (18). The conflicting results associating obesity with SCD in Iran are also reflected elsewhere, with strong associations among European and North American patients (10).

## Inherited cardiac ion channelopathies

**Figure 3:**
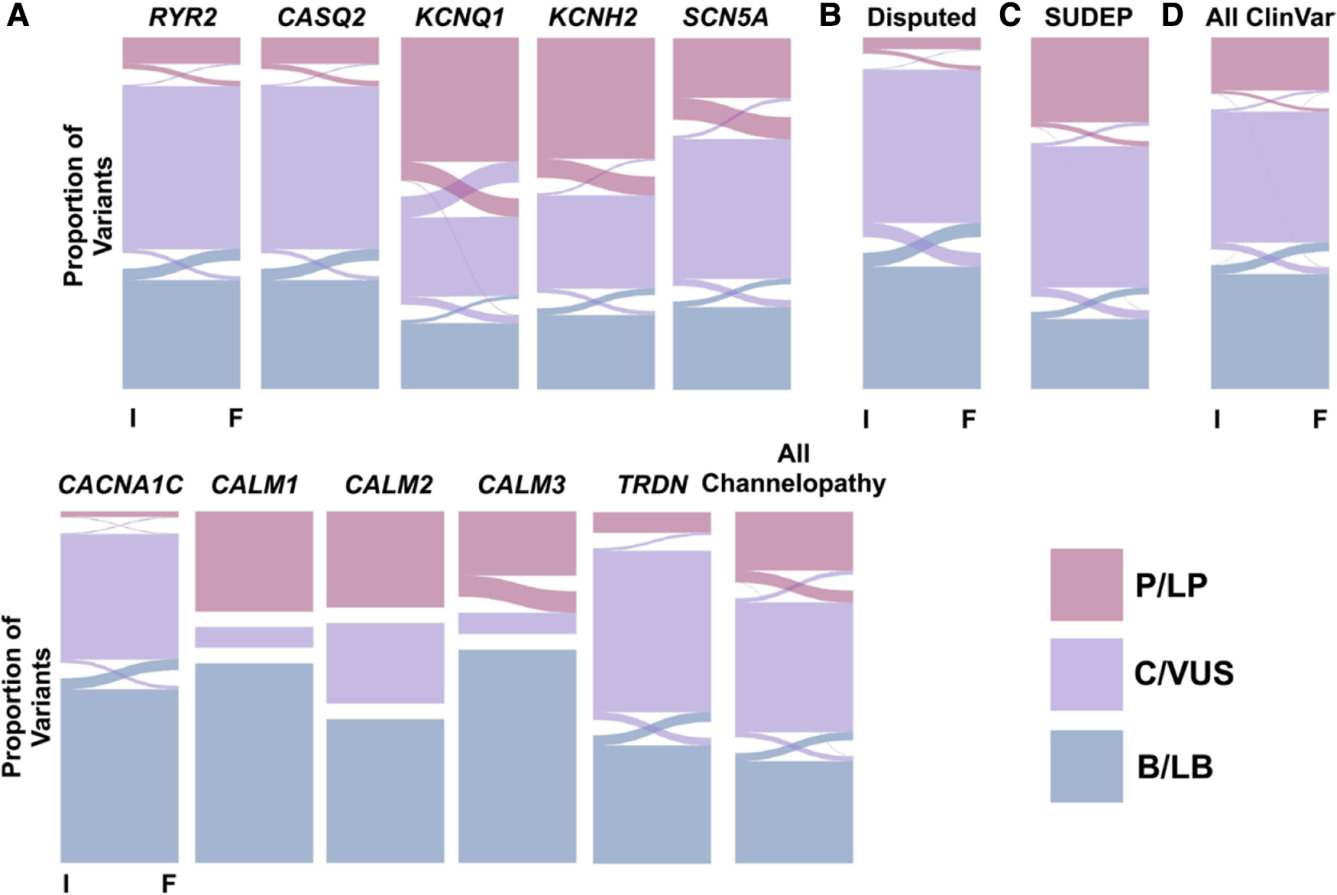
Alluvial plots presenting changes in variant evaluation from initial (**I**) to final (**F**) assessment in clinVar for variants that underwent at least two evaluations. Evaluations are categorized into pathogenic/likely pathogenic (P/LP, pink), conflicting interpretations of pathogenicity/variant of uncertain significance (C/VUS, purple), and benign/likely benign (B/LB, blue) buckets. Panel A shows gene-specific alluvial plots for cardiac channelopathy-associated genes. Panel B presents a summary alluvial plot for genes with an uncertain association with long QT syndrome. Panel C showcases a summary alluvial plot for genes linked to sudden unexpected death in epilepsy (SUDEP). Panel D provides a summary alluvial plot encompassing all gene variants in ClinVar. Reprinted from Rosamilia et al. (23) with permission.

Cardiac ion channelopathies can be acquired or inherited, with the latter being the focus of this review. Genetic variants that affect cardiac ion channels lead to hereditary channelopathies, and these changes can either cause gain or loss of ion channel function mechanisms (19). Cardiovascular channelopathies such as Brugada syndrome (BrS), long QT syndrome (LQTS), short QT syndrome (SQTS), and catecholaminergic polymorphic ventricular tachycardia (CPVT) are linked to SCD (Table 2) (5, 22). Given the significant presence of SCD in Asian populations and the relationship between ion channelopathies and SCD, it is imperative to review the particulars of each cardiac channelopathy specific to this ethnic group. Variants associated with channelopathies do not always have a straightforward connection between their pathogenicity and diagnostic certainty (23). Consequently, these variants are classified into different categories: benign/likely benign (B/LB), conflicting interpretations of pathogenicity/variant of uncertain significance (C/VUS), and pathogenic/likely pathogenic (P/LP). In a study conducted by Rosamilia et al., the researchers examined the frequency and directionality of significant changes in disease association (23). They found that 8.4% of variants in genes associated with channelopathies experienced a change in their pathogenicity status, leading to a decrease in the overall level of diagnostic certainty (Figure 3) (23).

**Table 2:** Summary of distinguishing features in cardiac ion channelopathies.

Cardiac ion channelopathy	Genes with strong evidence of association^b^	Ion channels affected	Clinical features presented
Brugada syndrome^a^	*SCN5A*	Na^+^ channel	Atypical right bundle branch block pattern. Cove-shaped ST-elevation in leads V1–V3. Can cause ventricular fibrillation and sudden cardiac death. Syncope may occur.
Long QT syndrome^a^	*KCNQ1*, *KCNH2, SCN5A, CALM1, CALM2, CALM3, TRDN, CACNA1C*^c^	K^+^ channels, Na^+^ channel, Ca^2+^ channel^c^	QT prolongation and slow ventricular repolarization. Can lead to torsade de pointes, syncope, and sudden cardiac death. Associated with Jervell and Lange-Nielsen syndrome as well as Romano-Ward syndrome.
Short QT syndrome^a^	*KCNQ1, KCNH2, KCNJ2*	K^+^ channels	QT shortening, early repolarization, tall T and U waves. Significantly associated with atrial fibrillation and ventricular arrhythmia.
Catecholaminergic polymorphic ventricular tachycardia^a^	*RYR2, CASQ2*	Ryanodine receptor	Can lead to ventricular tachycardia, ventricular fibrillation, and sudden cardiac death. Syncope may occur.

Table modified from Landstrom (20).

*SCN5A* (sodium voltage-gated channel alpha subunit 5), *KCNQ1* (potassium voltage-gated channel subfamily Q member 1), *KCNH2* (potassium voltage-gated channel subfamily H member 2), *CALM1* (calmodulin 1), *CALM2* (calmodulin 2), *CALM3* (calmodulin 3), *TRDN* (triadin), *CACNA1C* (calcium voltage-gated channel subunit alpha1 C), *KCNJ2* (potassium inwardly-rectifying channel subfamily J member 2), *RYR2* (ryanodine receptor 2), *CASQ2* (calsequestrin 2).

^a^ Genetic testing is recommended as a part of the diagnosis for all these channelopathies (21).

^b^ Based on ClinGen assessment of the strength of association with disease.

^c^ Moderate evidence.

## Brugada syndrome

BrS is a genetic cardiac condition that can be autosomal dominant or polygenic in inheritance (24). In this channelopathy, Na^+^ channels encoded by the *SCN5A* gene are affected by a loss-of-function mechanism, resulting in fatal ventricular arrhythmias and SCD (24). There have been reports of 23 genes linked to BrS (20, 25). Of these, *SCN5A* has the highest diagnostic yield (20%) in patients bearing the BrS phenotype and, thus, is the primary focus of genetic testing in BrS (20, 26). The pathogenicity of the remaining 22 genes remains inconclusive due to limited evidence (20, 26, 27). Genetic defects in the *SCN5A* gene cause 15%–30% of all cases with BrS, involving the *α*-subunit of the Na^+^ channel known as Na_v_1.5 (28). This channel is vital in generating action potentials and determining conductance velocity (29). Asians show a lower prevalence of *SCN5A* variants associated with BrS pathogenicity (30). For example, *SCN5A* variants are responsible for about 7.5%–8% of all BrS cases in Japanese and Taiwanese populations (31). While the general incidence of BrS is approximately 1 in 2000, Southeast Asian demographics display frequencies as high as 1 in 1,000 (29). In a nation-wide community-based study, the prevalence of BrS in Taiwan was estimated to be approximately 1 in 1,000, higher than Caucasians (32). While genetic testing is increasingly utilized, an electrocardiogram (ECG) remains the primary method of diagnosis (21). The characteristic symptom of BrS is an atypical right bundle branch block pattern with a cove-shaped ST-elevation in leads V1 to V3 (29).

BrS patients may experience aborted cardiac arrest or sustained ventricular tachycardia, which leads to an increased risk for future arrhythmias (33). Syncope is also often co-occurring with BrS at a rate of 30%, both at rest and during sleep, with fever being a noted trigger (29, 33). *SCN5A* variants involving changes to the pore region of the afflicted Na^+^ channel lead to a more severe phenotype and increased risk of severe cardiac events (33, 34). In particular, loss-of-function from *SCN5A* variants has been shown to be a significant predictor of SCD (34–36). BrS conventionally involves a significant reduction in Na^+^ current and delay in action potential conduction in the right ventricular outflow tract relative to the surrounding myocardium (33, 37). This often triggers ventricular tachycardia due to improper ion balance across the epicardium and endocardium barrier (33). BrS was initially characterized as a channelopathy. However, recent evidence showed normalization of the ECG pattern and reduced risk of arrhythmia following radiofrequency ablation treatment applied at the right ventricular outflow tract epicardium, suggesting that the pathogenesis may be due to electrical and structural abnormalities (33, 38). Although risk stratification has yet to be established, this reclassification might lead to new strategies for managing BrS (38, 39).

Although endemic to Southeast Asia, Ghaffari et al. studied 9 probands from Iran diagnosed with BrS (40). Alongside known polymorphisms, 20 novel variants were identified. This included 15 missense, 2 frameshift, 2 synonymous, and 1 nonsense variant (40). This study was added to the growing literature body from Asian countries to account for this syndrome's vast array of deleterious alleles. Despite Southeast Asia having the highest BrS burden, the majority of current research is from Western countries (41, 42). The most notable Asian multicenter study was performed in Japan, investigating the link between BrS and *SCN5A* variants (43). Results revealed that the Asian population with BrS were almost exclusively male adults with a higher frequency of aborted cardiac arrests and type 1 pattern (43). Given the sheer burden of this condition in Asia and the disproportionately low number of studies, more research on BrS risk factors and unique disease manifestations in the Asian population is warranted.

*SCN5A* variants have been linked to BrS due to the dysfunction of Na^+^ channel proteins, but the genetic etiologies of BrS remain unclear, especially in Asian populations. Nakajima et al. conducted a study investigating the role of *SCN5A* variants in the clinical presentation of BrS in Japanese patients (44). The study examined 30 subjects with a Brugada-pattern ECG, including 13 asymptomatic, 10 with a history of syncope, and 7 who were given cardiopulmonary resuscitation (44). Results identified 8 variants in *SCN5A* in these subjects, 6 of which were novel variants (44). This included a splice acceptor site variant (c.393-1c > t) in the syncope group that may result in a prematurely truncated protein and 4 novel missense variants in the asymptomatic group: A586T in domain 1 and domain 2 linker (D1-D2 linker) of Na^+^ channel protein, R689H (D1-D2 linker), S1553R between transmembrane segment 1 and 2 in domain 4 (S1-S2 in D4), and Q1706H (S5-Pore in D4). This demonstrates the importance of genetic variation in *SCN5A* regarding BrS, particularly relevant to Asian subpopulations such as the Japanese population (44).

In the first compound heterozygosity study of its type, Tan et al. examined the *SCN5A* gene and its relation to BrS in an Asian population (28). Tan et al. reported compound heterozygosity in the proband for SCN5A gene variants (p.A226V and p.R1629X), with each parent contributing one variant. Studies have shown that the greatest incidence of the gene is in Southeast Asian males (28). The average age of BrS onset is 41 ± 15 years. Thus, most individuals with these deleterious alleles pass on the variants to their offspring before exhibiting their own symptoms (28). *SCN5A* gene variants associated with BrS account for at least 4% of all SCDs. In another study by Makarawate et al., *SCN5A* genetic polymorphisms were studied for their association with increased defibrillator shocks in BrS (45). Over a span of 2 years, a total of 40 symptomatic BrS patients with a median age of 43 years and with active ICD implantations were observed, and 16 patients (40%) had appropriate ICD shock therapy after ICD treatment. The Na_v_1.5/R1193Q polymorphism was genetically associated with ICD shock therapy, presenting an adjusted hazard ratio of 10.550 (95% CI, 1.631–68.232) (45). The Na_v_1.5/R1193Q is a genetic marker that can cause defects in the electrophysiological pathways of the heart, leading to ventricular fibrillation in symptomatic BrS patients (45). Most notably, the p.A226V variant reduced peak Na^+^ current to 24% of the wildtype's output, while the p.R1629X variant resulted in a complete loss-of-function (28). Cardiac conduction delays are seen on ECGs, revealing longer PR interval, wider QRS complex, and increased frequency in BrS patients receiving ICD shock therapy than those without (45). This polymorphism is most frequently found in Asian populations, primarily Chinese (0.05–0.08), Vietnamese (0.08), and Japanese (0.02), as opposed to other ethnic cohorts, such as White Europeans (0.00) and Black Africans (0.00) (45).

A venue for future research involves looking at racial/ethnic differences in BrS for Asian pediatric patients. A recent study that included 11 Asian and 40 Caucasian patients showed that pediatric patients (age < 12) that have experienced arrhythmogenic events are more prone to developing BrS than adolescents. However, it should be noted that the study did not compare differences between races/ethnicities (46). Greater care needs to be taken when developing a treatment for younger patients, a sensitive demographic. While ICD and defibrillator therapy has shown moderate success, careful and precise technique is required to avoid inappropriate shock treatment and other complications (47).

Overall, Asian populations face a higher incidence of BrS than White populations, despite fewer patients displaying pathogenic *SCN5A* variants (30). Instead, Asian populations appear more vulnerable to BrS through environmental interactions and oligogenic risk (30, 48). Asian patients with *SCN5A* variants are predisposed to major arrhythmic events (49, 50). There are genetic differences between Asian and White BrS patients (30). For example, an *SCN5A* promoter region haplotype associated with increased electrical abnormalities was present in Japanese patients while absent in White and Black patients (51). Additionally, several single nucleotide polymorphisms regularly found in White and Japanese BrS patients were found to be prolific in Taiwanese BrS patients (30). Therefore, it appears that Asian populations show different genetic features related to risk stratification of BrS compared to other ethnicities, possibly warranting a different clinical approach (30, 39, 52). Thus, these genetic differences between Asian and White patients suggest the need for a distinct clinical approach in managing BrS.

## Long QT syndrome

**Figure 4:**
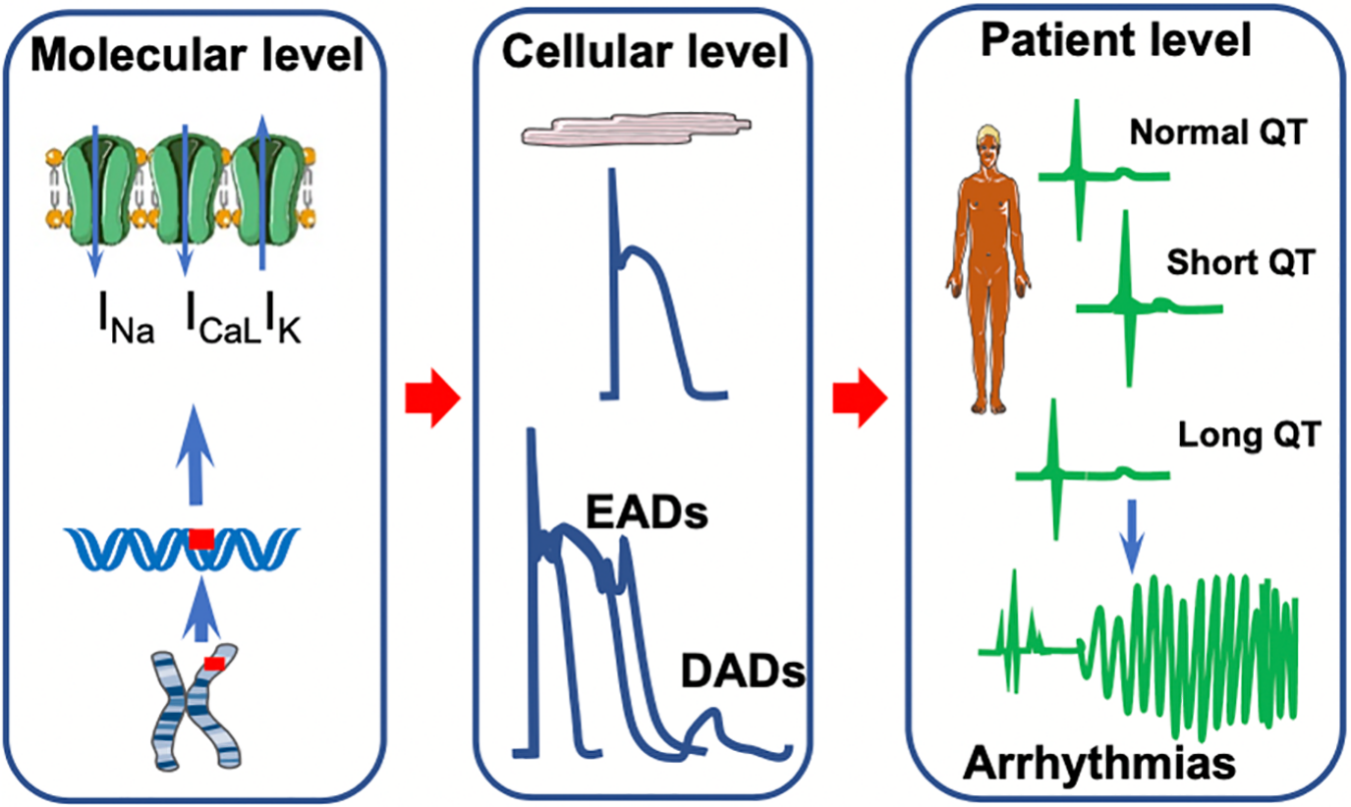
Summary of clinical and physiological features of cardiac ion channelopathies. A schematic representation of the molecular, cellular, and patient levels of channelopathies related to short and long QT syndromes. At the molecular level, gene variants affect ion currents in Na^+^, Ca^2+^, and K^+^ channels. This, in turn, affects the action potential morphology by forming early (EADs) and/or delayed after depolarizations (DADs) at the cellular level. These electrical abnormalities translate into either a short or long QT interval on the surface electrocardiogram predisposing to arrhythmia at the patient level.

Channelopathies are a group of diseases caused by ion channel subunit dysfunction and their interacting proteins (Figure 4). LQTS is one of the most prominent disorders under this umbrella term, being extensively studied on a clinical and genetic level (5, 22). It is an inherited arrhythmia associated with prolonged ventricular repolarization and fatal cardiac events such as SCD. Variants in ion channel genes leading to channelopathies are associated with an increased risk of developing LQTS (53). One such gene associated with LQTS is *KCNH2* which codes for the human ether à-go-go related gene (hERG) ion channel, and the variants are part of the LQT2 genotype (53). QT prolongation and slowed ventricular repolarization occur via the loss-of-function of K^+^ channels and the gain-of-function of Na^+^ channels (53). The increased QT interval can lead to a polymorphic form of ventricular tachycardia known as torsade de pointes, ultimately resulting in syncope and SCD (53). LQT1, LQT2, and LQT3 are distinct subtypes of LQTS, with LQT1 caused by variants in the *KCNQ1* gene, LQT2 caused by variants in the *KCNH2* gene, and LQT3 caused by variants in the *SCN5A* gene, highlighting different affected ion channels and underlying mechanisms contributing to prolonged QT interval and arrhythmia susceptibility (54). In patients with pathogenic LQTS gene variants, 80%–85% show the LQT1 and LQT2 genotypes leading to K^+^ channel dysfunction, while 5%–13% of patients show the LQT3 genotype that leads to abnormal cardiac Na^+^ channel function (55). The association between LQTS genotypes and disease severity was identified in 1998 by Zareba et al., and in 2003 the same group demonstrated a significant relationship between age, sex, genotype, and LQTS progression in patients (55). In the past decade, a collaboration between various international research groups further demonstrated the relationship between the LQTS genotype and clinical phenotype (55). However, these studies were done primarily in Caucasian Western populations, and more work is to be done in populations of other ethnicities, such as Asians, in which the LQTS disease burden is significant (55).

Although LQTS has been observed in every ethnic group, the polymorphism variations set them apart. *KCNH2* A915V polymorphisms were primarily seen in Asian populations (4.5%) (56). In one study by Akimoto et al., two Japanese families with LQTS underwent genomic analysis of the *KCNH2* gene in search of potential variants (57). The medical histories, genomic data, and ECGs for 5 members from Family 1 and 7 members from Family 2 were analyzed for *KCNH2* variants. The results showed that LQTS members of Family 1 had significantly reduced T-wave amplitudes compared to unaffected members (0.16 ± 0.77 vs. 0.35 ± 0.05 mV, *P* < 0.01) (57). The study also found a new missense variant of *KCNH2* in Family 1 at codon 601 in the extracellular loop between the S5 and pore domains (57). Single-strand conformation analysis confirmed that this missense variant was not a rare polymorphism; it was present in 3 LQTS family members but not in any unaffected individuals nor 100 other reference genomes from healthy individuals (57). This was a breakthrough discovery of a novel missense variant in *KCNH2*, with possible broader implications for the Japanese and Asian populations (57).

It has previously been suggested that there are variations between the clinical presentations and outcomes of LQTS in adult and pediatric populations. In a retrospective cohort study, Lee et al. measured the development of spontaneous ventricular fibrillation and ventricular tachycardia in patients from public Hong Kong hospitals (58). A total of 142 patients aged 27 ± 23 years at symptom onset were examined (58). The results showed that the patient history of ventricular fibrillation and ventricular tachycardia (HR 4.67, 95% CI 1.53–14.3 *P* = 0.007), Schwartz score (HR = 1.90, 95% CI 1.11–3.26, *P* = 0.020), and non-ventricular fibrillation and non-ventricular tachycardia arrhythmias (HR = 5.41, 95% CI 1.36–21.4, *P* = 0.016) were predictive of the development of spontaneous ventricular fibrillation and ventricular tachycardia and that Schwartz score was predictive for adults alone (HR = 4.67, 95% CI 1.48–14.7, *P* = 0.009) (58). This demonstrates a few clinical differences in LQTS presentation in adults compared to children in an Asian subpopulation, previously unknown (58).

There are 17 common genes associated with LQTS development, of which only 7 genes have definitive evidence of being linked to this disorder (20). Genotype-specific ECG patterns associated with LQT1-3 are present in variant carriers (59). Gao et al. evaluated the role of ECG-guided gene studies in a large-scale genotyping study using a cohort of Chinese LQTS patients (59). The study examined 230 Chinese patients (26 ± 17 years, 66% female) using a genotype prediction method in 200 patients based on ECG patterns associated with each genotype (85 LQT1, 110 LQT2, and 5 LQT3) (59). Family members of the probands were also examined similarly (59). Results revealed LQT2 as the most common genotype in the Chinese cohort studied. Further genetic screening showed 104 variants (46 *KCNQ1*, 54 *KCNH2*, and 4 *SCN5A* variants) in the probands (59). This ECG-guided genotyping had a predictive accuracy of 79% (157/200) overall, 79% (67/85) for LQT1, 78% (86/110) for LQT2, and 80% (4/5) for LQT3. ECG-guided genotyping also demonstrated a predictive accuracy of 98% (42/43) in the family members (58, 59). This highlights the utility and cost-saving potential of cross-referencing common ECG patterns with common LQTS genes (59).

**Figure 5:**
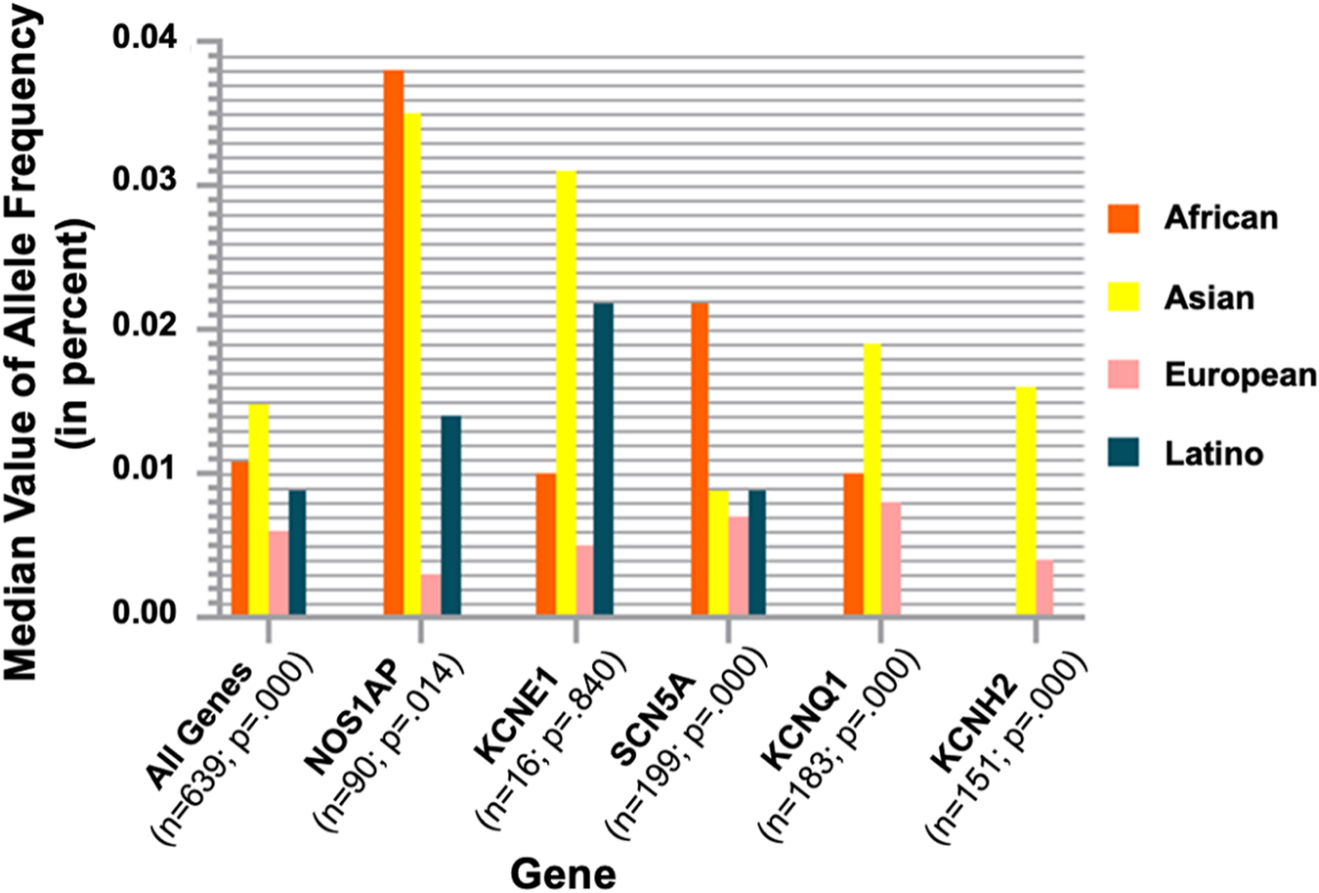
Ethnic group gene analysis from the exome aggregation consortium database. For all genes related to ion channelopathies, the number of alleles (*n*), the *P* values, and the median values of allele frequencies across different ethnic groups are shown. Asian populations had the highest proportion of allele frequencies in *KCNE1*, *KCNQ1*, and *KCNH2*. Reprinted from Kong et al. (60) with permission.

LQTS is a preventable cause of SCD in several different populations. In LQTS, ventricular repolarization is marked by prolongation of the corrected QT interval (QTc) on ECG, and it is a detectable and preventable cause of SCD in young populations (53, 56). There is usually a causative genetic variant with a large effect size identified in 80% of the probands (genotype-positive) (53). However, in several LQTS cases, genetic studies fail to identify such a variant with a large effect size. As such, common genetic variations of smaller effect sizes associated with LQTS may be more significant. Lahrouchi et al. conducted a genome-wide association study and transethnic meta-analysis, examining single-nucleotide polymorphisms in 1,656 patients of European and Japanese ancestry with LQTS and 9,890 controls (53). They assessed for common variant heritability in LQTS and calculated the overall effect of the identified polymorphisms associated with the QT interval using a polygenic risk score in the general population. This study identified three loci associated with LQTS near *NOS1AP*, *KCNQ1*, and *KLF12* (*P* < 5 × 10^−8^) and 1 missense variant in *KCNE1* (p.Asp85Asn) (*P* < 10^−6^) (53). The analysis demonstrated that approximately 15% of the variation in LQTS susceptibility could be due to common genetic variation (heritability-SNP 0.148; standard error 0.019). The polygenic risk score of QT-interval-associated common genetic variants was higher in LQTS cases than in controls (*P* < 10^−13^) and higher in genotype-negative vs. genotype-positive patients (53). This demonstrates the role of common genetic variation in the clinical LQTS, especially in genotype-negative patients. Furthermore, in a meta-analysis, Kong et al. assessed the mean allele frequencies of channelopathy genes *SCN5A*, *KCNH2*, *KCNE1*, *KCNQ1*, and *NOS1AP* in Black, White, Asian, and Hispanic subjects. The findings indicated that while Asian populations possessed a higher number of gene alleles associated to SCD, not all of these variants were associated with channelopathy or increased risks of SCD (Figure 5) (60).

**Figure 6:**
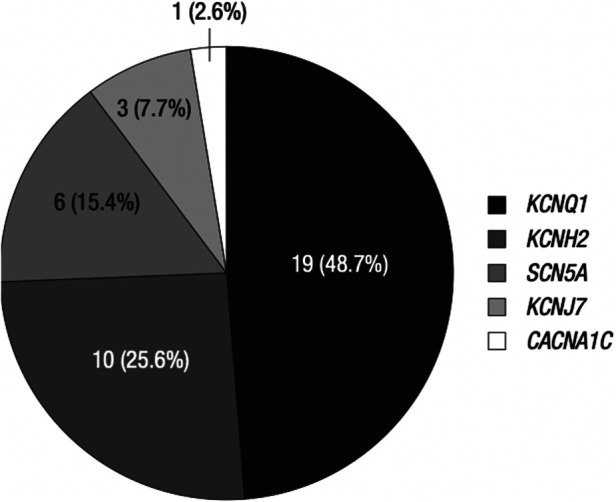
Genetic variants in Korean proband and family group. The studied genes are *KCNQ1*, *KCNH2*, *SCN5A*, KCNJ7, and *CACNA1C*, corresponding to LQT1, 2, 3, 7, and 8, respectively. *KCNQ1* and *KCNH2* together account for almost 75% of all genetic variants in the Korean ethnic group. Only 2.6% of the genetic variants were related to *CACNA1C*. Reprinted from Lee et al. (61) with permission.

While genetic factors related to LQTS have been widely studied, there have been very few clinical and genetic studies in the Korean demographic. Lee et al. conducted a large single-center study investigating 62 LQTS patients and 19 family members with an LQTS gene variant (61). The clinical presentations in the proband group were ECG abnormalities (38.7%), aborted cardiac arrest (24.2%), and syncope or seizure (19.4%) (61). Genetic variants were found in 40 of the 61 cases studied (Figure 6) (61). They found variants of *KCNQ1* in 19, *KCNH2* in 10, *SCN5A* in 7, *KCNJ2* in 3, and *CACNA1C* in 1 (61). In the LQTS patients, QTc prolongation was significantly associated with syncope (*P* = 0.001), ventricular tachycardia (*P* = 0.017) and aborted arrest (*P* = 0.010) (61). *β*-blocker therapy significantly reduced QTc in these patients (*P* = 0.007). The variants in the family members showed a penetrance of 57.9% (61).

Variants in *KCNQ1*, *KCNH2*, and *SCN5A* account for approximately 90% of the genotypes of LQTS patients (62). These variants result in LQT1, 2, and 3 phenotypes, respectively (62). Of note, the LQT1 phenotype is associated with symptoms during exercise, LQT2 is associated with emotional stress, and LQT3 is marked by symptoms during sleep (62). Chen et al. conducted a study to identify a variant in a Japanese patient with QT prolongation and sinus node dysfunction on cardiac exercise and epinephrine stress tests, indicative of an LQT3 phenotype (63). The study found a heterozygous missense *SCN5A* variant V2016M in cardiac Na^+^ channels resulting in a loss of function with reduced Na^+^ current peak densities (WT: 175.2 ± 17.6 pA/pF vs. V2016M 97.2 ± 16.0 pA/pF; *P* < .01) (63). The variant also caused a gain-of-function marked by increased late Na^+^ currents through protein kinase A activation (WT: 0.07 ± 0.01% vs. V2016M 0.17 ± 0.03%; *P* < .05) (63). These variants in *SCN5A* combined with exercise-epinephrine-induced symptoms demonstrate an unusual genotypic-phenotypic association. However, this association is observed only in isolated cases and is therefore atypical in Asians (63–65). There is value in genetic testing before cardiac diagnosis (21). For example, patients with variants that encode for the pore regions in the hERG channel are more likely to present severe cardiac phenotypes than other variants (22, 66).

There are additional conditions associated either with LQTS itself or genetic markers of the disease. Jervell and Lange-Nielsen syndrome is an autosomal recessive condition associated with variants in the K^+^ channel gene *KCNQ1* (LQTS1). It is also characterized by congenital deafness (67). However, additional genetic studies are required to identify the main disease-causing variants and other rare genes leading to the progress of Jervell and Lange-Nielsen syndrome. Schulze-Barr et al. conducted a haplotype analysis of microsatellite markers in a Lebanese family with this condition but did not detect a linkage at the LQTS1 gene (67). The analysis of this family excluded two other ion-channel genes involved in autosomal dominant LQTS, *KCNH2* (LQTS2) and *SCN5A* (LQTS3) (67). These findings demonstrate that Jervell and Lange-Nielsen syndrome is genetically heterogeneous and that an unknown LQTS gene or even common genetic variation could contribute to this condition's overall phenotype in this family. Another related condition is Romano-Ward syndrome, an autosomal-dominant form of LQTS that can lead to SCD secondary to tachyarrhythmia (68). Lee-Chen et al. examined genetic factors related to LQTS in a Taiwanese family. Genetic analysis revealed a variant in the *KCNH2* gene (68). The group amplified the coding regions and exon-intron borders of the *KCNH2* gene and used single-strand conformation polymorphism analysis to identify variants (68). A *KCNH2* exon with an unusual pattern was cloned and sequenced, eventually revealing a C to T replacement in codon 614, leading to the substitution of valine for alanine in the *KCNH2* protein's pore region (68). Further analysis showed that this variant was present in all affected family members. This reflects the value of preventative genetic screening, which could be used to identify disease-causing LQTS variants that can facilitate early treatment. Thus, LQTS appears to be poorly studied in the Asian population, resulting in underrepresentation in the scientific literature. While there seem to be risk factors and markers for the disease unique to Asian subpopulations, there is not enough information to accurately judge the risk LQTS represents to this ethnic group.

L-type Ca^2+^ channel variants are known to be linked to the development of different inherited cardiac arrhythmias, including BrS and LQTS (Timothy syndrome) (69, 70). However, these variants have not been widely studied in Asian populations (69). Fukuyama et al. examined the frequency of these variants in a Japanese population and compared them to *SCN5A* variant carriers (64). The study cohort consisted of 312 probands registered in 2 Japanese institutes between 1996 and 2012 (64). The subjects in the cohort had different cardiac arrhythmias, including BrS and idiopathic ventricular fibrillation, as per clinical symptoms and ECG findings (64). The cohort was also screened for *CACNA1C* and *CACNB2b* variants. They compared the clinical attributes between probands with gene variants in *CACNA1C* or *SCN5A*, as there were no *CACNB2b* variants in the cohort (64). Results showed 6 *CACNA1C* variants in 7 unrelated probands and *SCN5A* variants in 20 probands (64). Half of the variants were located in the C-terminus. Among 7 *CACNA1C* variant carriers, 2 were female, and 3 showed clinical symptoms; 2 patients were resuscitated from ventricular fibrillation, and 1 patient had syncope (64). There were no significant differences in ECG findings between *CACNA1C* carriers and *SCN5A* carriers, illustrating that while the variant frequency of *CACNA1C* may be low, it may still be clinically relevant to perform L-type Ca^2+^ channel variant screening to prevent life-threatening cardiac events (64). Collectively, differences between Asian populations and White populations in relation to LQTS include a higher prevalence of specific polymorphisms, distinct clinical presentations, and varied genotypic-phenotypic associations. These differences highlight the importance of considering ethnic diversity in LQTS research and clinical management.

## Short QT syndrome

SQTS can be a potential cause of tachyarrhythmia and SCD while also being associated with autonomic changes and ionic imbalances such as hyperkalemia and hypercalcemia. Funada et al. sought to study the prevalence of SQTS and assess overall QTc distribution in a large-single center study at a Japanese university hospital (71). The study enrolled 12,149 subjects who received an ECG between 2003 and 2004, of which 10,984 subjects had their most recent ECGs studied with a focus on corrected QTc (71). The QTc values analyzed appeared very similar to a normal distribution (408 ± 25 msec^1/2^), with females (412 ± 24 msec^1/2^) having significantly longer (*P* < 0.05) QTc values than males. Among 5,511 males, 69 (1.25%) showed QTc <354 msec^1/2^ (2 standard deviations below the mean in males), and among 5,473 females, 89 subjects (1.63%) exhibited QTc <364 msec^1/2^ (2 standard deviations below the mean in females) (71). Only 3 subjects (0.03% in all subjects and 0.05% in males) exhibited QTc <300 msec^1/2^; however, none had clinical symptoms of SQTS (71). This shows that while LQTS is quite prevalent in Asian populations, SQTS may be much more uncommon. As such, it is necessary to study at some level of depth for treatment to be available when patients are affected.

SQTS, although rare, also presents differently across several different demographics. Specifically, the Korean population is associated with SQTS and short QT interval (SQTI). In a large multicenter study, Kim et al. assessed the long-term effects and clinical prognosis of SQTS in consecutive Korean patients at three university hospitals between 1999 and 2019 (72). SQTI was defined as a QT interval of ≤ 340 msec in serial ECGs. In this study, 34 SQTI patients were followed for an average of 4.8 years. Patients with normal QTc were age and sex-matched and included in a 1:4 ratio for the study (72). The clinical features of patients with and without SQTI were analyzed. Results showed that tall T waves, U waves, and early repolarization (*P* < 0.001) were significantly more frequent in SQTI subjects than in those without (72). QT dispersion was significantly wider [44.0 (28.0–73.0) vs. 20.0 (12.0–35.0) msec, *P* < 0.001], and heart rate was significantly decreased in SQTI patients compared to those without [52.0 (47.0–58.0) vs. 70.0 (62.3–84.0)/min, *P* < 0.001] (72). Atrial fibrillation (11.8% vs. 2.2%, *P* = 0.030) and ventricular arrhythmia (8.7% vs. 0%, *P* = 0.007) were also significantly more frequent in the SQTI patients as opposed to those without. Atrial fibrillation and SQTS share a common variant association with the *KCNQ1* gene (72). Overall, SQTI was significantly related to both atrial fibrillation and ventricular arrhythmia with an odds ratio of 5.911 at the 95% confidence interval [1.257–27.808; *P* = 0.025] (72). Thus, SQTS presents in this Asian subpopulation with associated potentially fatal arrhythmias.

The use of inconsistent QT interval cutoffs makes the SQTS diagnosis difficult clinically (73). In a large study (*N* = 114,334) by Miyamoto et al., SQTS intervals were found to be more frequent in males, showed bimodal peaks at both ends of the age spectrum, and demonstrated a strong relationship to the development of AF (73). A study by Nikoo et al. assessed SQTI prevalence in an Iranian subset of a larger cohort of 4,363 adult subjects in which the QT interval on ECGs was analyzed for bradycardia, signs of early repolarization, atrial fibrillation, and other arrhythmias (74). The SQTS susceptibility group showed a higher predominance of males and a lower average heart rate compared to the normal QT interval group (M/F of 1/0.26 vs. 1/1.145, *P*-value < 0.0001; 58 ± 9 vs. 71 ± 12; *P*-value < 0.0001) (74). Two subjects with a high probability of developing SQTS and three with an intermediate likelihood of attaining it were identified (74). Frequency of atrial fibrillation, syncope, bradycardia, early repolarization, low voltage ECG, and infantile SCD in first- and second-degree relatives were 16.67, 4.17, 33.33, 11.11, 11.11, 11.11%, respectively (74). This showed that a higher frequency of cardiac events and the development of arrhythmias are associated with increased SQTS-susceptibility within the cohort (74). In a combined manner, differences between Asian populations and White populations regarding SQTS can be observed. While SQTS appears to be more uncommon in Asian populations compared to LQTS, SQTS in Asian subpopulations is associated with potentially fatal arrhythmias such as atrial fibrillation and ventricular arrhythmia, indicating a distinct clinical profile.

## Catecholaminergic polymorphic ventricular tachycardia

Catecholaminergic polymorphic ventricular tachycardia (CPVT) is an inherited arrhythmia aggravated by stress and physical exertion. The genetic basis of this condition has been widely studied in Western populations, but the role of variants in CPVT-related genes is still unclear in the Asian demographic (75). The ryanodine receptor 2 (*RYR2*) gene is implicated in CPVT but may also be involved in other cardiovascular diseases. *RYR2* is an ion channel that plays a crucial role in Ca^2+^ release from the myocardial sarcoplasmic reticulum, whose dysfunction or aberrant expression can lead to fatal ventricular arrhythmia and SCD. In a genomic sequencing study, Kawamura et al. examined genetic variants in 3 CPVT-related genes: *RYR2*, calsequestrin 2 (*CASQ2*), and inward-rectifier K^+^ channel 2 (*KCNJ2*) in 50 unrelated Japanese CPVT-diagnosed probands as well as designated *RYR2* genotyped and non-genotyped groups (76). They also performed the same testing on 46 Japanese family members (76). The study's results showed 28 *RYR2* variants (56.0% of total variants found), 1 compound heterozygous *CASQ2* (2.0%) variant, and 1 *KCNJ2* (2.0%) variant (76). Moreover, the *RYR2* variant carrier group showed increased bidirectional ventricular tachycardia on ECGs and increased use/access of *β*-blockers for treatment compared to the group without *RYR2* variants (76).

In another Japanese study by Aizawa et al., 83 Japanese patients with idiopathic ventricular fibrillation, LQTS, BrS, CPVT, or arrhythmogenic right ventricular cardiomyopathy underwent genetic testing of *RYR2* to find disease-causing variants (77). The results showed 3 *RYR2* gene variants in 4 families with CPVT and no significant association with other arrhythmias (77). This implies that *RYR2* is more significantly linked to CPVT than other arrhythmias and highlights the genetic basis of CPVT in the Japanese population (77). Overall, CPVT should be of particular interest when considering treatment and prevention within the Japanese population. Further research is required to determine its significance and genetic markers in other Asian subpopulations.

Wang et al. conducted a case-control study to determine the potential role of a 4-base pair (4-bp) indel polymorphism (rs10692285) in the 3′UTR region of *RYR2* with relation to the risk of SCD by coronary heart disease in a Chinese population (78). Logistic regression analysis showed that the insertion allele of rs10692285 was significantly associated with increased risk of SCD (OR = 2.03; 95% CI = 1.08–3.77; *P* = 0.0161; power = 0.743) (78). Genotypic analysis revealed increased insertion allele expression at the mRNA and protein levels vs. the deletion allele. Furthermore, the Dual-Luciferase assay demonstrated significantly higher transcription activity on *RYR2* with this polymorphism in the ins/ins genotype compared to the del/del genotype (78). This highlights the potential role of genotypic studies and screening for polymorphisms related to the risk of SCD. In another study, Akilzhanova et al. tested for variants at common disease-associated *RYR2* locus in 35 Kazakhstani patients with ventricular arrhythmia episodes; two had characteristics of CPVT, and 33 patients had monomorphic idiopathic ventricular arrhythmia (75). Results showed two novel variants at *RYR2* locus, including one de-novo variant (c13892A > T; p.D4631V) in a CPVT patient and a rare novel variant (c5428G > C; p.V1810l) of significance in a patient with idiopathic ventricular tachycardia (75). A known variant previously associated with arrhythmogenic right ventricular dysplasia type-2 was also identified. This study demonstrates the potential pathogenic associations of novel missense variants with the development of life-threatening ventricular arrhythmias and SCD, thereby showing the utility of creating predictive tests in areas where genotype data is uncommon (75, 78). Taken together, differences between Asian populations and White populations regarding CPVT are evident. The genetic basis of CPVT and the associated variants in CPVT-related genes, such as *RYR2*, demonstrate differences between these populations, highlighting the importance of understanding the distinct clinical profile and genetic markers of CPVT in Asian subpopulations.

## Conclusion

**Figure 7:**
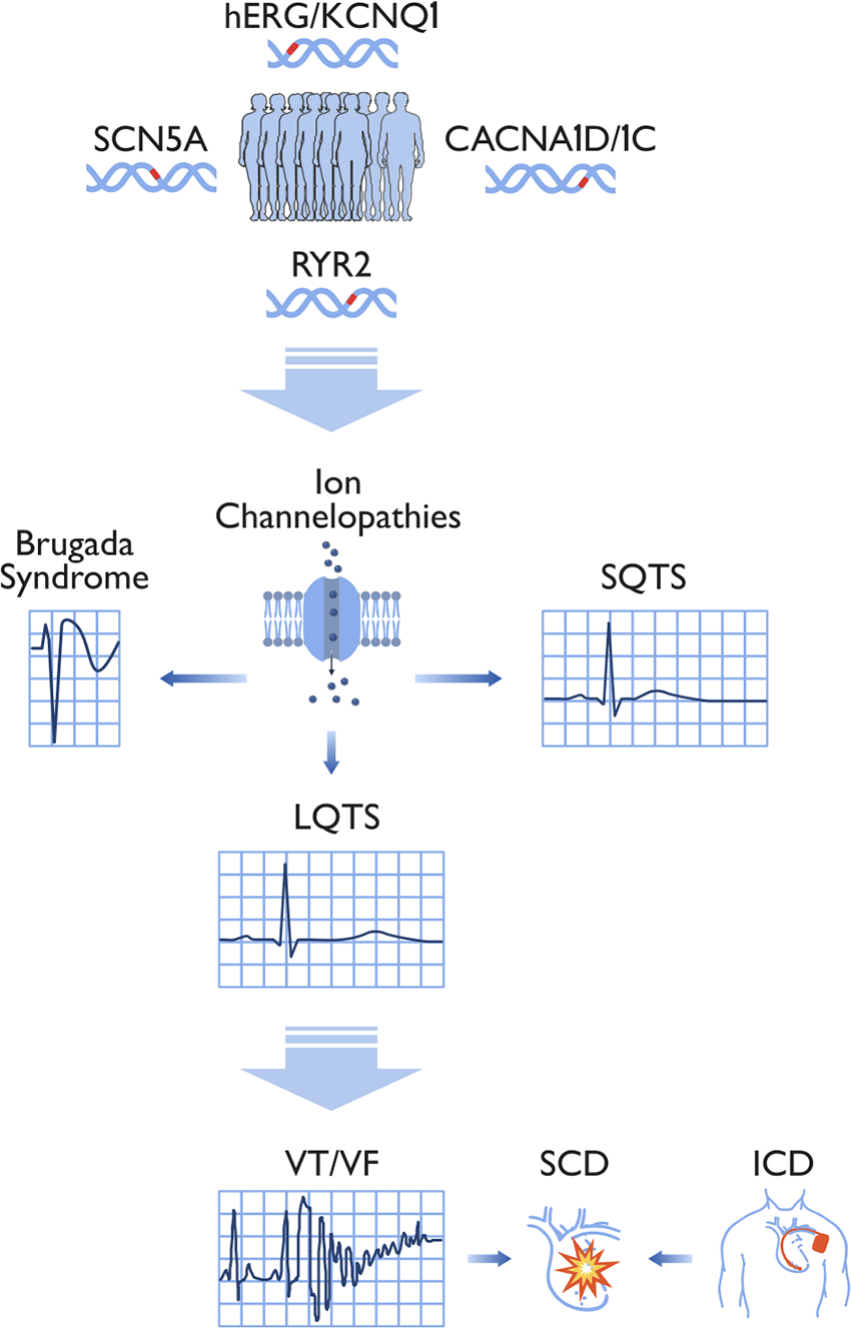
Associations between significant genes, ion channelopathies, sudden cardiac death, and device therapy. This illustration displays the links between representative genes susceptible to variants (hERG /*KCNQ1*, *SCN5A*, *CACNA1C*, and *RYR2*), ion channelopathies, and sudden cardiac death (SCD) in the Asian population. Brugada syndrome (BrS) and long QT syndrome (LQTS) were identified as the most significant channelopathies in the Asian population. These channelopathies can lead to atrial fibrillation (AF) and/or ventricular tachycardia (VT) and ventricular fibrillation (VF). Asian SCD patients do not receive implantable cardioverter-defibrillator (ICD) treatment compared to the White population in the United States. hERG (human ether à-go-go related gene); *KCNQ1* (potassium voltage-gated channel subfamily Q member 1); *SCN5A* (sodium voltage-gated channel alpha subunit 5); *CACNA1C* (calcium voltage-gated channel subunit alpha 1C); *RYR2* (ryanodine receptor 2).

Differences in risks, disease prevalence, and treatment exist between Asian and Western populations for ion channelopathies and SCD. Asians are genetically prone to various arrhythmias and channelopathies, such as BrS and LQTS (Figure 7). It is evident that Asians have unique risk factors concerning heart disease, which warrants special attention when designing methodologies, pharmacological treatments, and standard guidelines to diagnose and treat Asian populations. Moreover, there is a paucity of data regarding the use and effectiveness of treatments such as ICDs to counter SCD in Asian populations.

At the same time, there are positives to take away from this review. The sheer volume of literature available for review is a strong indicator that professionals in the healthcare industry are increasingly aware of the racial and ethnic differences presented for rhythm disturbances in Asian and other populations, such as Black/African Americans. Some studies showed no significant differences in the diseases between Asian and Western populations. However, this can be attributed to the fact that the race and ethnicity aspects of arrhythmias have been overlooked and that the body of research concerning certain arrhythmias in Asian populations is still growing. Overall, encouraging attempts are being made to reduce these gaps, making research, diagnostic methods, and treatment options more inclusive and improving outcomes related to SCD and ion channelopathies in Asian populations.

**Figure 8:**
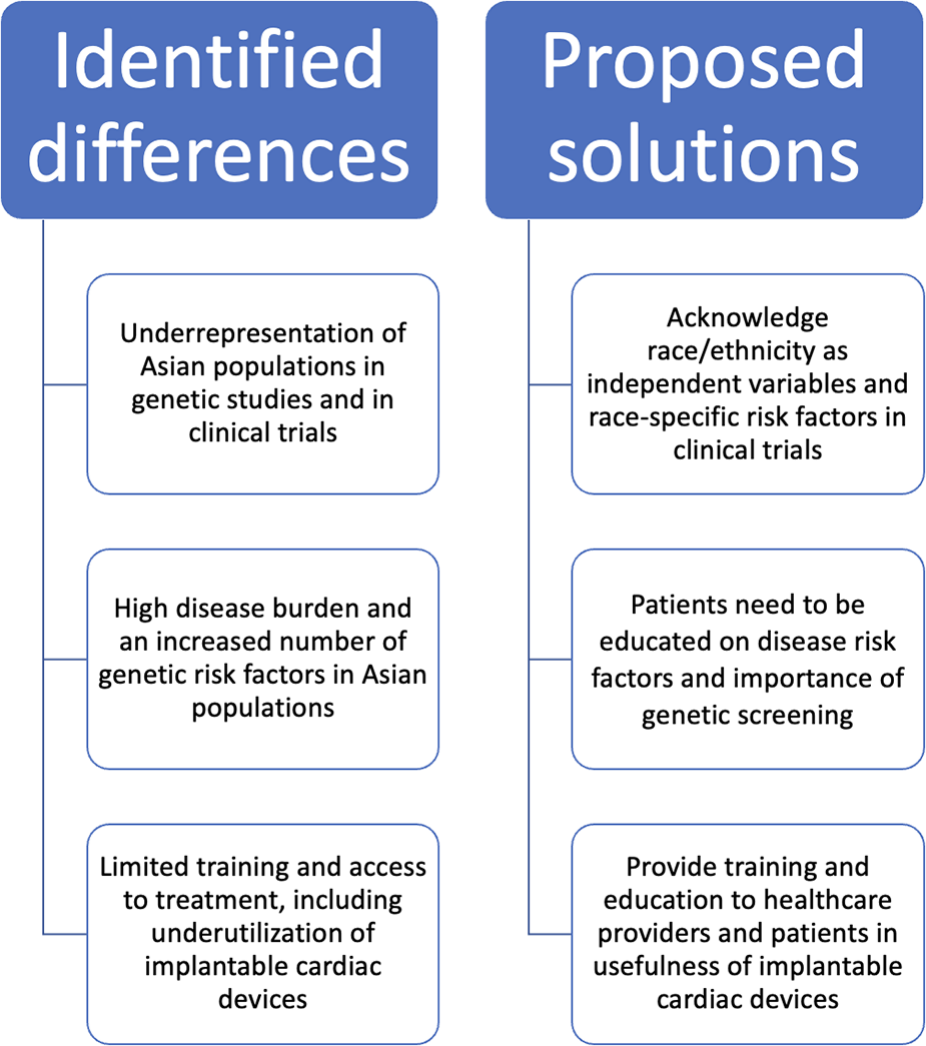
Summary of differences in Asian populations and proposed solutions. Identified differences in Asian populations and potential solutions are illustrated. The inadequate inclusion of race/ethnicity in clinical studies and limited training and education for healthcare providers and patients may contribute to differences in ion channelopathies in Asian populations. Solutions include broader enrollment of Asian populations in clinical trials, access to cardiac devices, and health education on sudden cardiac death risk factors.

To address differences between Asians and other ethnicities, we first suggest acknowledging race/ethnicity and the associated risk factors in medical research subjects, clinical trials, and hospital treatment. Training can be made available to physicians and researchers alike to enhance awareness of the differences and provide knowledge on the genetic risk factors Asians present regarding SCD and ion channelopathies. Screening for BrS and LQTS in Asian subpopulations is likely to be a useful preventative measure in improving outcomes, given the prevalence of these diseases. Education on these conditions and associated risks should also be made available to the Asian subpopulations, as well as what can be done to lower risk and what treatments can be sought once the relevant conditions are active in an individual. In this way, the prevention of disease, speed of diagnosis, and treatment can all be improved, leading to more positive outcomes in clinics for Asian patients (Figure 8).

## References

[1] Chahine M Fontaine JM Boutjdir M. Racial disparities in Ion channelopathies and inherited cardiovascular diseases associated with sudden cardiac death. J Am Heart Assoc. (2022) 11(6):e023446. 10.1161/jaha.121.023446 35243873 PMC9075281

[2] Bureau USC. (2021). Race and Ethnicity in the United States: 2010 Census and 2020 Census. Available at: https://www.census.gov/library/visualizations/interactive/race-and-ethnicity-in-the-united-state-2010-and-2020-census.html

[3] Lewsey SC Breathett K. Racial and ethnic disparities in heart failure: current state and future directions. Curr Opin Cardiol. (2021) 36(3):320–8. 10.1097/hco.0000000000000855 33741769 PMC8130651

[4] Shah NS Kandula NR. Addressing Asian American misrepresentation and underrepresentation in research. Ethn Dis. (2020) 30(3):513–6. 10.18865/ed.30.3.513 32742157 PMC7360176

[5] Murakoshi N Aonuma K. Epidemiology of arrhythmias and sudden cardiac death in Asia. Circ J. (2013) 77(10):2419–31. 10.1253/circj.cj-13-1129 24067274

[6] Zhang S. Sudden cardiac death in China: current status and future perspectives. Europace. (2015) 17(Suppl 2):ii14–18. 10.1093/europace/euv143 26842111

[7] Feng XF Hai JJ Ma Y Wang ZQ Tse HF. Sudden cardiac death in mainland China: a systematic analysis. Circ Arrhythm Electrophysiol. (2018) 11(11):e006684. 10.1161/circep.118.006684 30571181

[8] Wu G Liu J Wang S Yu S Zhang C Wang D N-terminal pro-brain natriuretic peptide and sudden cardiac death in hypertrophic cardiomyopathy. Heart. (2021) 107(19):1576–83. 10.1136/heartjnl-2020-317701 33361398

[9] Huang PS Cheng JF Ko WC Chang SH Lin TT Chen JJ Unique clinical features and long term follow up of survivors of sudden cardiac death in an Asian multicenter study. Sci Rep. (2021) 11(1):18250. 10.1038/s41598-021-95975-8 34521870 PMC8440502

[10] Toreyhi H Asgari S Khalili D Pishgahi M Azizi F Hadaegh F. Sudden cardiac death among Iranian population: a two decades follow-up of Tehran lipid and glucose study. Sci Rep. (2021) 11(1):15720. 10.1038/s41598-021-95210-4 34344986 PMC8333266

[11] Cruz-Flores S Rabinstein A Biller J Elkind MS Griffith P Gorelick PB Racial-ethnic disparities in stroke care: the American experience: a statement for healthcare professionals from the American heart association/American stroke association. Stroke. (2011) 42(7):2091–116. 10.1161/STR.0b013e3182213e24 21617147

[12] Stiles MK Wilde AAM Abrams DJ Ackerman MJ Albert CM Behr ER 2020 APHRS/HRS expert consensus statement on the investigation of decedents with sudden unexplained death and patients with sudden cardiac arrest, and of their families. Heart Rhythm. (2021) 18(1):e1–e50. 10.1016/j.hrthm.2020.10.010 33091602 PMC8194370

[13] Fishman GI Chugh SS Dimarco JP Albert CM Anderson ME Bonow RO Sudden cardiac death prediction and prevention: report from a national heart, lung, and blood institute and heart rhythm society workshop. Circulation. (2010) 122(22):2335–48. 10.1161/circulationaha.110.976092 21147730 PMC3016224

[14] Tseng ZH Olgin JE Vittinghoff E Ursell PC Kim AS Sporer K Prospective countywide surveillance and autopsy characterization of sudden cardiac death: pOST SCD study. Circulation. (2018) 137(25):2689–700. 10.1161/circulationaha.117.033427 29915095 PMC6013842

[15] Ueda N Noda T Kusano K Yasuda S Kurita T Shimizu W. Use of implantable cardioverter-defibrillators for primary prevention of sudden cardiac death in Asia. JACC Asia. (2023) 3(3):335–45. 10.1016/j.jacasi.2023.02.004 37323866 PMC10261895

[16] Shah NS Xi K Kapphahn KI Srinivasan M Au T Sathye V Cardiovascular and cerebrovascular disease mortality in Asian American subgroups. Circ Cardiovasc Qual Outcomes. (2022) 15(5):e008651. 10.1161/circoutcomes.121.008651 35535589 PMC9117444

[17] Jose PO Frank AT Kapphahn KI Goldstein BA Eggleston K Hastings KG Cardiovascular disease mortality in Asian Americans. J Am Coll Cardiol. (2014) 64(23):2486–94. 10.1016/j.jacc.2014.08.048 25500233 PMC4274749

[18] Goldstein SA Li S Lu D Matsouaka RA Rymer J Fonarow GC Implantable cardioverter defibrillator utilization and mortality among patients ≥65 years of age with a low ejection fraction after coronary revascularization. Am J Cardiol. (2021) 138:26–32. 10.1016/j.amjcard.2020.09.056 33068540

[19] Kim YG Oh SK Choi HY Choi JI. Inherited arrhythmia syndrome predisposing to sudden cardiac death. Korean J Intern Med. (2021b) 36(3):527–38. 10.3904/kjim.2020.481 33092314 PMC8137412

[20] Landstrom AP Kim JJ Gelb BD Helm BM Kannankeril PJ Semsarian C Genetic testing for heritable cardiovascular diseases in pediatric patients: a scientific statement from the American heart association. Circ Genom Precis Med. (2021) 14(5):e000086. 10.1161/hcg.0000000000000086 34412507 PMC8546375

[21] Wilde AAM Semsarian C Márquez MF Shamloo AS Ackerman MJ Ashley EA European heart rhythm association (EHRA)/heart rhythm society (HRS)/Asia pacific heart rhythm society (APHRS)/latin American heart rhythm society (LAHRS) expert consensus statement on the state of genetic testing for cardiac diseases. Europace. (2022) 24(8):1307–67. 10.1093/europace/euac030 35373836 PMC9435643

[22] Schwartz PJ Ackerman MJ Antzelevitch C Bezzina CR Borggrefe M Cuneo BF Inherited cardiac arrhythmias. Nat Rev Dis Primers. (2020) 6(1):58. 10.1038/s41572-020-0188-7 32678103 PMC7935690

[23] Rosamilia MB Lu IM Landstrom AP. Pathogenicity assignment of variants in genes associated with cardiac channelopathies evolve toward diagnostic uncertainty. Circ Genom Precis Med. (2022) 15(3):e003491. 10.1161/circgen.121.003491 35543671

[24] Marsman EMJ Postema PG Remme CA. Brugada syndrome: update and future perspectives. Heart. (2022) 108(9):668–75. 10.1136/heartjnl-2020-318258 34649929

[25] Juang JJ Horie M. Genetics of brugada syndrome. J Arrhythm. (2016) 32(5):418–25. 10.1016/j.joa.2016.07.012 27761167 PMC5063259

[26] Zeppenfeld K Tfelt-Hansen J de Riva M Winkel BG Behr ER Blom NA 2022 ESC guidelines for the management of patients with ventricular arrhythmias and the prevention of sudden cardiac death. Eur Heart J. (2022) 43(40):3997–4126. 10.1093/eurheartj/ehac262 36017572

[27] Hosseini SM Kim R Udupa S Costain G Jobling R Liston E Reappraisal of reported genes for sudden arrhythmic death: evidence-based evaluation of gene validity for brugada syndrome. Circulation. (2018) 138(12):1195–205. 10.1161/circulationaha.118.035070 29959160 PMC6147087

[28] Tan BY Yong RY Barajas-Martinez H Dumaine R Chew YX Wasan PS A brugada syndrome proband with compound heterozygote SCN5A mutations identified from a Chinese family in Singapore. Europace. (2016) 18(6):897–904. 10.1093/europace/euv058 25829473

[29] Vohra J Rajagopalan S. Update on the diagnosis and management of brugada syndrome. Heart Lung Circ. (2015) 24(12):1141–8. 10.1016/j.hlc.2015.07.020 26412486

[30] Milman A Behr ER Gray B Johnson DC Andorin A Hochstadt A Genotype-phenotype correlation of SCN5A genotype in patients with brugada syndrome and arrhythmic events: insights from the SABRUS in 392 probands. Circ Genom Precis Med. (2021) 14(5):e003222. 10.1161/circgen.120.003222 34461752

[31] Jimmy Juang JM Liu YB Julius Chen CY Yu QY Chattopadhyay A Lin LY Validation and disease risk assessment of previously reported genome-wide genetic variants associated with brugada syndrome: SADS-TW BrS registry. Circ Genom Precis Med. (2020) 13(4):e002797. 10.1161/circgen.119.002797 32490690 PMC7439932

[32] Juang JM Chen CY Chen YH Wu IC Hsu CC Chen LN Prevalence and prognosis of brugada electrocardiogram patterns in an elderly han Chinese population: a nation-wide community-based study (HALST cohort). Europace. (2015) 17(Suppl 2):ii54–62. 10.1093/europace/euv141 26842116

[33] Malik BR Ali Rudwan AM Abdelghani MS Mohsen M Khan SHA Aljefairi N Brugada syndrome: clinical features, risk stratification, and management. Heart Views. (2020) 21(2):88–96. 10.4103/heartviews.Heartviews_44_20 33014301 PMC7507903

[34] O'Neill MJ Wada Y Hall LD Mitchell DW Glazer A Roden DM. Functional assays reclassify suspected splice-altering variants of uncertain significance in Mendelian channelopathies. Circ Genom Precis Med. (2022) 15(6):101161circgen122003782. 10.1161/circgen.122.003782 PMC9772980 36197721

[35] Baroudi G Pouliot V Denjoy I Guicheney P Shrier A Chahine M. Novel mechanism for brugada syndrome: defective surface localization of an SCN5A mutant (R1432G). Circ Res. (2001) 88(12):E78–83. 10.1161/hh1201.093270 11420310

[36] Ishikawa T Kimoto H Mishima H Yamagata K Ogata S Aizawa Y Functionally validated SCN5A variants allow interpretation of pathogenicity and prediction of lethal events in brugada syndrome. Eur Heart J. (2021) 42(29):2854–63. 10.1093/eurheartj/ehab254 34219138

[37] Joviano-Santos JV Santos-Miranda A Neri EA Fonseca-Alaniz MH Krieger JE Pereira AC SCN5A Compound heterozygosity mutation in brugada syndrome: functional consequences and the implication for pharmacological treatment. Life Sci. (2021) 278:119646. 10.1016/j.lfs.2021.119646 34048814

[38] Pieroni M Notarstefano P Oliva A Campuzano O Santangeli P Coll M Electroanatomic and pathologic right ventricular outflow tract abnormalities in patients with brugada syndrome. J Am Coll Cardiol. (2018) 72(22):2747–57. 10.1016/j.jacc.2018.09.037 30497561

[39] Nakano Y Shimizu W. Brugada syndrome as a major cause of sudden cardiac death in asians. JACC Asia. (2022) 2(4):412–21. 10.1016/j.jacasi.2022.03.011 36339362 PMC9627855

[40] Ghaffari T Mirhosseini Motlagh N Daraei A Tafrihi M Saravi M Sabour D. Novel SCN5A variants identified in a group of Iranian brugada syndrome patients. Funct Integr Genomics. (2021) 21(3–4):331–40. 10.1007/s10142-021-00778-9 33641026

[41] Kalra A Kumbhani DJ Hill JA. Cardiovascular science India tour: impacting cardiovascular disease in south Asia. Circulation. (2020) 141(3):159–60. 10.1161/circulationaha.119.043837 31958245

[42] Lee S Zhou J Li KHC Leung KSK Lakhani I Liu T Territory-wide cohort study of brugada syndrome in Hong Kong: predictors of long-term outcomes using random survival forests and non-negative matrix factorisation. Open Heart. (2021b) 8:1. 10.1136/openhrt-2020-001505 PMC7871343 33547222

[43] Yamagata K Horie M Aiba T Ogawa S Aizawa Y Ohe T Genotype-phenotype correlation of SCN5A mutation for the clinical and electrocardiographic characteristics of probands with brugada syndrome: a Japanese multicenter registry. Circulation. (2017) 135(23):2255–70. 10.1161/circulationaha.117.027983 28341781

[44] Nakajima T Kaneko Y Saito A Irie T Tange S Iso T Identification of six novel SCN5A mutations in Japanese patients with brugada syndrome. Int Heart J. (2011) 52(1):27–31. 10.1536/ihj.52.27 21321465

[45] Makarawate P Chaosuwannakit N Vannaprasaht S Sahasthas D Koo SH Lee EJD SCN5A Genetic polymorphisms associated with increased defibrillator shocks in brugada syndrome. J Am Heart Assoc. (2017) 6:6. 10.1161/jaha.116.005009 PMC5669154 28584071

[46] Michowitz Y Milman A Andorin A Sarquella-Brugada G Gonzalez Corcia MC Gourraud JB Characterization and management of arrhythmic events in young patients with brugada syndrome. J Am Coll Cardiol. (2019) 73(14):1756–65. 10.1016/j.jacc.2019.01.048 30975291

[47] Gonzalez Corcia MC Sieira J Pappaert G de Asmundis C Chierchia GB La Meir M Implantable cardioverter-defibrillators in children and adolescents with brugada syndrome. J Am Coll Cardiol. (2018) 71(2):148–57. 10.1016/j.jacc.2017.10.082 29325638

[48] Monasky MM Micaglio E Ciconte G Pappone C. Brugada syndrome: oligogenic or mendelian disease? Int J Mol Sci. (2020) 21(5):1687. 10.3390/ijms21051687 32121523 PMC7084676

[49] Rattanawong P Chenbhanich J Mekraksakit P Vutthikraivit W Chongsathidkiet P Limpruttidham N SCN5A Mutation status increases the risk of major arrhythmic events in Asian populations with brugada syndrome: systematic review and meta-analysis. Ann Noninvasive Electrocardiol. (2019) 24(1):e12589. 10.1111/anec.12589 30126015 PMC6931443

[50] Cerrone M Costa S Delmar M. The genetics of brugada syndrome. Annu Rev Genomics Hum Genet. (2022) 23:255–74. 10.1146/annurev-genom-112921-011200 35567276

[51] Bezzina CR Shimizu W Yang P Koopmann TT Tanck MW Miyamoto Y Common sodium channel promoter haplotype in Asian subjects underlies variability in cardiac conduction. Circulation. (2006) 113(3):338–44. 10.1161/circulationaha.105.580811 16415376

[52] Chen C Tan Z Zhu W Fu L Kong Q Xiong Q Brugada syndrome with SCN5A mutations exhibits more pronounced electrophysiological defects and more severe prognosis: a meta-analysis. Clin Genet. (2020) 97(1):198–208. 10.1111/cge.13552 30963536

[53] Lahrouchi N Tadros R Crotti L Mizusawa Y Postema PG Beekman L Transethnic genome-wide association study provides insights in the genetic architecture and heritability of long QT syndrome. Circulation. (2020) 142(4):324–38. 10.1161/circulationaha.120.045956 32429735 PMC7382531

[54] Mazzanti A Maragna R Vacanti G Monteforte N Bloise R Marino M Interplay between genetic substrate, QTc duration, and arrhythmia risk in patients with long QT syndrome. J Am Coll Cardiol. (2018) 71(15):1663–71. 10.1016/j.jacc.2018.01.078 29650123

[55] Zareba W. Sex and genotype in long QT syndrome risk stratification. JAMA Cardiol. (2019) 4(3):254–5. 10.1001/jamacardio.2018.4947 30758500

[56] Modell SM Lehmann MH. The long QT syndrome family of cardiac ion channelopathies: a HuGE review. Genet Med. (2006) 8(3):143–55. 10.1097/01.gim.0000204468.85308.86 16540748

[57] Akimoto K Furutani M Imamura S Furutani Y Kasanuki H Takao A Novel missense mutation (G601S) of HERG in a Japanese long QT syndrome family. Hum Mutat. (1998) Suppl 1:S184–186. 10.1002/humu.1380110159 9452080

[58] Lee S Zhou J Jeevaratnam K Wong WT Wong ICK Mak C Paediatric/young versus adult patients with long QT syndrome. Open Heart. (2021a) 8:2. 10.1136/openhrt-2021-001671 PMC8438947 34518285

[59] Gao Y Liu W Li C Qiu X Qin X Guo B Common genotypes of long QT syndrome in China and the role of ECG prediction. Cardiology. (2016) 133(2):73–8. 10.1159/000440608 26496715

[60] Kong T Feulefack J Ruether K Shen F Zheng W Chen XZ Ethnic differences in genetic Ion channelopathies associated with sudden cardiac death: a systematic review and meta-analysis. Ann Clin Lab Sci. (2017) 47(4):481–90. 28801377

[61] Lee YS Kwon BS Kim GB Oh SI Bae EJ Park SS Long QT syndrome: a Korean single center study. J Korean Med Sci. (2013) 28(10):1454–60. 10.3346/jkms.2013.28.10.1454 24133349 PMC3792599

[62] Tester DJ Ackerman MJ. Genetic testing for potentially lethal, highly treatable inherited cardiomyopathies/channelopathies in clinical practice. Circulation. (2011) 123(9):1021–37. 10.1161/circulationaha.109.914838 21382904 PMC3073829

[63] Chen J Makiyama T Wuriyanghai Y Ohno S Sasaki K Hayano M Cardiac sodium channel mutation associated with epinephrine-induced QT prolongation and sinus node dysfunction. Heart Rhythm. (2016) 13(1):289–98. 10.1016/j.hrthm.2015.08.021 26282245

[64] Fukuyama M Ohno S Wang Q Kimura H Makiyama T Itoh H L-type calcium channel mutations in Japanese patients with inherited arrhythmias. Circ J. (2013) 77(7):1799–806. 10.1253/circj.cj-12-1457 23575362

[65] Sasaki T Ikeda K Nakajima T Kawabata-Iwakawa R Iizuka T Dharmawan T Multiple arrhythmic and cardiomyopathic phenotypes associated with an SCN5A A735E mutation. J Electrocardiol. (2021) 65:122–7. 10.1016/j.jelectrocard.2021.01.019 33610078

[66] Isbister JC Raju H. Genetic testing for inherited heart disease in the era of next-generation sequencing: now, next, and beyond. Circ Genom Precis Med. (2022) 15(5):101161circgen122003925. 10.1161/circgen.122.003925 36173694

[67] Schulze-Bahr E Haverkamp W Wedekind H Rubie C Hördt M Borggrefe M Autosomal recessive long-QT syndrome (jervell lange-nielsen syndrome) is genetically heterogeneous. Hum Genet. (1997) 100(5–6):573–6. 10.1007/s004390050554 9341873

[68] Lee-Chen GJ Tai DY Chu CH Teng YN. Romano-ward long QT syndrome: identification of a HERG mutation in a Taiwanese kindred. J Formos Med Assoc. (1999) 98(9):649–52. 10560244

[69] Splawski I Timothy KW Sharpe LM Decher N Kumar P Bloise R Ca(V)1.2 calcium channel dysfunction causes a multisystem disorder including arrhythmia and autism. Cell. (2004) 119(1):19–31. 10.1016/j.cell.2004.09.011 15454078

[70] Delinière A Haddad C Herrera-Siklódy C Hermida A Pruvot E Bressieux-Degueldre S Phenotypic characterization of timothy syndrome caused by the CACNA1C p.Gly402Ser variant. Circ Genom Precis Med. (2023) 16(3):280–2. 10.1161/circgen.122.004010 37009738

[71] Funada A Hayashi K Ino H Fujino N Uchiyama K Sakata K Assessment of QT intervals and prevalence of short QT syndrome in Japan. Clin Cardiol. (2008) 31(6):270–4. 10.1002/clc.20208 18543308 PMC6653181

[72] Kim DY Uhm JS Kim M Kim IS Jin MN Yu HT Long-term prognosis of short QT interval in Korean patients: a multicenter retrospective cohort study. BMC Cardiovasc Disord. (2021a) 21(1):17. 10.1186/s12872-020-01824-3 33407155 PMC7788900

[73] Miyamoto A Hayashi H Yoshino T Kawaguchi T Taniguchi A Itoh H Clinical and electrocardiographic characteristics of patients with short QT interval in a large hospital-based population. Heart Rhythm. (2012) 9(1):66–74. 10.1016/j.hrthm.2011.08.016 21855519

[74] Nikoo MH Heiran A Mashayekh F Rezaianzadeh A Shiravani A Azadian F. A descriptive report on short QT interval in Kherameh branch of the PERSIAN cohort study. Sci Rep. (2022) 12(1):2898. 10.1038/s41598-022-06835-y 35190598 PMC8861052

[75] Akilzhanova A Guelly C Nuralinov O Nurkina Z Nazhat D Smagulov S RYR2 Sequencing reveals novel missense mutations in a Kazakh idiopathic ventricular tachycardia study cohort. PLoS One. (2014) 9(6):e101059. 10.1371/journal.pone.0101059 24978818 PMC4076244

[76] Kawamura M Ohno S Naiki N Nagaoka I Dochi K Wang Q Genetic background of catecholaminergic polymorphic ventricular tachycardia in Japan. Circ J. (2013) 77(7):1705–13. 10.1253/circj.cj-12-1460 23595086

[77] Aizawa Y Mitsuma W Ikrar T Komura S Hanawa H Miyajima S Human cardiac ryanodine receptor mutations in ion channel disorders in Japan. Int J Cardiol. (2007) 116(2):263–5. 10.1016/j.ijcard.2006.02.024 16843546

[78] Wang S Zhang Z Yang Y Wang C Tao R Hu S An insertion/deletion polymorphism within 3′UTR of RYR2 modulates sudden unexplained death risk in Chinese populations. Forensic Sci Int. (2017) 270:165–72. 10.1016/j.forsciint.2016.12.005 27987400

